# The mammalian INO80 chromatin remodeling complex is required for replication stress recovery

**DOI:** 10.1093/nar/gku605

**Published:** 2014-07-12

**Authors:** Ivelina Vassileva, Iskra Yanakieva, Michaela Peycheva, Anastas Gospodinov, Boyka Anachkova

**Affiliations:** Institute of Molecular Biology, Bulgarian Academy of Sciences, Academy G. Bonchev St. 21, 1113 Sofia, Bulgaria

## Abstract

A number of studies have implicated the yeast INO80 chromatin remodeling complex in DNA replication, but the function of the human INO80 complex during S phase remains poorly understood. Here, we have systematically investigated the involvement of the catalytic subunit of the human INO80 complex during unchallenged replication and under replication stress by following the effects of its depletion on cell survival, S-phase checkpoint activation, the fate of individual replication forks, and the consequences of fork collapse. We report that INO80 was specifically needed for efficient replication elongation, while it was not required for initiation of replication. In the absence of the Ino80 protein, cells became hypersensitive to hydroxyurea and displayed hyperactive ATR-Chk1 signaling. Using bulk and fiber labeling of DNA, we found that cells deficient for Ino80 and Arp8 had impaired replication restart after treatment with replication inhibitors and accumulated double-strand breaks as evidenced by the formation of γ-H2AX and Rad51 foci. These data indicate that under conditions of replication stress mammalian INO80 protects stalled forks from collapsing and allows their subsequent restart.

## INTRODUCTION

During DNA replication, genome integrity is particularly vulnerable, since various factors—such as chemical agents, proteins tightly bound to DNA or specific DNA structures—could act as obstacles and stall advancing replication forks. If not restarted, stalled forks collapse and produce double-strand breaks and these fork-associated DNA lesions are a major source of genome instability in cancer development (1–3). In eukaryotes DNA is organized into chromatin. The basic unit of chromatin is the nucleosome, which is composed of 147 bp of DNA wrapped around a histone octamer comprising a tetramer of (H3–H4)_2_ flanked by two dimers of H2A–H2B. During replication the chromatin structure undergoes major reorganization as nucleosomes are disassembled ahead of the replication fork and reassembled behind it. An increasing body of evidence suggests that replicative helicases, histone chaperones and chromatin remodelers form an assembly line at the replication forks (4). This necessitates the study of the contribution of ATP-dependent chromatin remodeling complexes in the processes of chromatin replication and maintenance of genome stability (4–6).

INO80 is an ATP-dependent chromatin remodeling complex composed of 15 subunits in yeast (7) and 13 in humans (8). A recent study has provided the architectural framework of the yeast complex and its interaction with the nucleosome. The INO80 remodeler possesses a specific elongated embryo-shaped head-neck-body-foot structure in which the nucleosome is sandwiched between the head and the foot, the latter being conformationally flexible and able to promote nucleosome remodeling. (9). In functional terms, the INO80 complex has been shown to participate in various nuclear processes, including transcriptional regulation (10–12), double-strand break repair (13–16) and nucleotide excision repair (17,18). It has been linked with the maintenance of the chromatin structure of centromeres (19) and telomeres (20), as well as with sister chromatid cohesion (21) and chromosome segregation (22). A number of studies done mostly in yeast have implicated the INO80 chromatin remodeler in replication. It has been shown that when cells enter S phase Ino80 is recruited to a significant portion of the yeast autonomous replication sequences and their vicinities (23–25). The yeast INO80 complex has been implicated to play a role when normal fork progression is impeded, yet different studies have generated dissimilar results. Thus, inhibition of replication induced by hydroxyurea (HU) in Ino80 deletion mutant led to dissociation of Polα, RPA (Replication Protein A) and Mcm4 from chromatin, suggesting that Ino80 had a crucial role in stabilizing stalled replication forks to ensure their proper restart (25). In line with these findings, other investigators found that Ino80 mutants treated with HU displayed significantly delayed or impaired resumption of DNA synthesis and accumulation of Rad52 foci, suggesting on-going homologous recombination (HR) repair of broken forks (23). Conversely, Falbo et al. (24) reported that while INO80 is necessary for the resumption of replication at forks stalled by methyl methane sulfonate, it is not required for replication fork stabilization after treatment with HU, indicating the involvement of the complex in DNA damage tolerance during S phase. While in an earlier report it was shown that Ino80 was not required for checkpoint activation in response to replication stress (25), a later study reported a novel role for the Ino80 and Isw2 chromatin remodelers in DNA replication. The authors showed that the remodelers attenuated and deactivated the S-phase checkpoint signaling in parallel with the Rad53 phosphatases pathway (26).

So far, the data about the role of Ino80 in replication in higher eukaryotes has been rather incomplete. The knockdown of human Ino80 resulted in slower growth and reduced rate of S-phase progression (22). A very recent report (27) found that the knockout of mammalian Ino80 led to HU sensitivity, defective telomere replication and impaired HR repair at telomeres. The limited data about the impact of the INO80 complex on replication and replication stress recovery in higher eukaryotes and the discrepancies in yeast studies necessitate a systematic examination of INO80 involvement in these processes. To this end, we examined the impact of depletion of subunits of the mammalian INO80 complex on the initiation and elongation steps of replication, as well as the resumption of stalled replication forks. We found that Ino80-depleted cells had reduced rate of replication elongation, but fired more replication origins to complete S phase. These cells were hypersensitive to HU and displayed elevated Chk1 signaling. A large fraction of replication forks in Ino80 and Arp8-deficient cells failed to restart after release from replication inhibition. Knocked-down cells accumulated γ-H2AX and Rad51 foci, suggesting on-going HR repair of collapsed replication forks. Thus, for the first time in mammalian cells, our data provide evidence that the INO80 chromatin remodeler is required for fork progression and maintenance of stalled replication forks.

## MATERIALS AND METHODS

### Cell culture, treatment and esiRNA knockdown

Human PC3 cells were grown in Dulbecco's modified Eagle's medium supplemented with 10% fetal bovine serum, 1 mM pyruvate and antibiotics in 5% CO_2_ atmosphere. To induce replication stress cells were treated with either HU (0.5 mM, unless otherwise indicated) or 2 μM aphidicolin. For synchronization the cells were first treated with 2.5 mM thymidine for 18 h, released in fresh medium for 6 h, treated with 25 ng/ml nocodazole for 12 h and subjected to mitotic shake-off. Mitotic cells were washed twice and released in fresh medium for 12 h to enter S phase.

EsiRNAs targeting the coding regions of human Ino80 (3440–3894, transcript NM_017553.1), human Arp8 (485–916, transcript NM_022899.3) or GFP (Green Fluorescent Protein) (132–591) were synthesized as previously described (28,29). Primers used to amplify the targeted regions were selected using Riddle database (30):

hIno80 (5′-TCACTATAGGGAGAGTGTGGAGCATCAGACCTCAG; 5′-CACTATAGGGAGACCCTGCTTTGTCTGCCCTAAG).

hArp8 (5′-TCACTATAGGGAGAGGGCACGCTCCTACAATAAGC; 5′-TCACTATAGGGAGACGTGCTGCTTAAGCCACTTCC).

Quantities of Lipofectamine and esiRNAs for efficient knockdown were optimized using esiRNA against Eg5 (Kif11). Typically 60 pmol of esiRNA and 2 μl of Lipofectamine 2000 were used per well in a 24 well plate (500 μl transfection volume). Knockdown of Ino80 was assessed by western blotting. Under these conditions the level of the targeted proteins were routinely decreased by more than 70%. Primers to assess transcript level of human Arp8 were 5′-TGATGGCCGGCAACGATTCCG and 5′-TTCCATGCAATCAGCCGGGGG.

### Western blots and biochemical fractionation

To prepare total protein lysates, cells were collected with a cell scraper, washed twice in phosphate-buffered saline (PBS), boiled in Laemmli sample buffer (50 mM Tris-HCl, pH 6.8, 100 mM β-mercaptoethanol, 1% sodium dodecyl sulphate (SDS), 10% glycerol, 0.0025% Bromphenol blue) and sonicated to shear DNA. Alternatively, cells were lysed in buffer containing 50 mM Tris-HCl, pH 7.4, 150 mM NaCl, 0.5% Triton X-100, 0.1% SDS, 1 mM Ethylenediaminetetraacetic acid (EDTA), protease and phosphatase inhibitors, sonicated, and spin-clarified at 15 000 x g for 10 min, mixed with Laemmli sample buffer (to a final concentration of 1x of the latter) and boiled. Lysates were resolved on sodium dodecyl sulphate-polyacrylamide gel electrophoresis gels (6 or 12.5%, as appropriate) according to Laemmli (31), and blotted on nitrocellulose membrane (BioRad). Equal loading and transfer were monitored by Ponceau S staining of the membranes and by actin or H2B immunostaining.

Fractionation was carried out essentially as described in (32). Cells were washed twice with PBS, scraped and re-suspended in buffer A (10 mM HEPES, pH 7.9, 10 mM KCl, 1.5 mM MgCl_2_, 0.34 M sucrose, 10% glycerol, 1 mM dithiothreitol and protease inhibitors) at about 3–4 × 10^7^ cells/ml. Triton X-100 was added to a final concentration of 0.1%, and the cells were incubated for 5 min on ice. The fraction of soluble proteins was separated from the chromatin fraction by centrifugation (5 min, 1250 x *g*). The chromatin fraction was washed in buffer B (3 mM EDTA, 0.2 mM EGTA, 1 mM dithiothreitol, protease inhibitors), re-suspended in the same buffer and sonicated.

Primary antibodies used were rabbit anti-Ino80 antibody (Novus), rabbit anti-phospho-Chk1 (Cell Signaling technologies), mouse anti-RPA32 (Abcam), mouse anti-actin (Abcam) and rabbit anti-histone H2B (Santa Cruz Biotechnologies). Proteins were visualized with HRP-conjugated anti-rabbit or anti-mouse IgG (1:5000; Santa Cruz Biotechnologies) and ECL chemiluminiscence system (Novex) or using Li-cor Odyssey IR imaging system with appropriate IRDye-labeled secondary antibodies (Li-cor Biosciences).

### Flow cytometry, survival assay and determination of the rate of DNA synthesis

To analyze cell cycle profiles, cells were harvested by trypsinization and fixed in 70% ethanol. Before analysis cells were re-suspended in PBS, treated with RNAse A (20 μg/ml) and stained with propidium iodide (20 μg/ml). Analysis was carried out by a FACScalibur apparatus with Cellquest software (Becton Dickinson). To assess clonogenic survival, mock or Ino80-silenced cells (72 h after esiRNA transfection) were seeded at 500 cells/cm2 and treated with the indicated concentrations of HU for 48 h. After 10 days of growth in fresh medium to form visible colonies, they were washed, fixed with acetic acid/methanol (1 vol:7 vol) and stained with 0.5% gentian violet. To determine the rate of DNA synthesis, DNA was labeled uniformly by incubating the cells overnight with 0.025 μCi/ml of [^14^C]-thymidine (dT) (50 mCi/mmol; Du Pont). The newly synthesized DNA after treatment with HU was pulse-labeled for 30 min with 1 μCi/ml of [^3^H]-dT (30 mCi/mmol; Amersham). The cells were trypsinized and precipitated with trichloroacetyc acid on GF/C filters (Millipore). Radioactivity was measured using Beckman scintillation counter. The rate of DNA synthesis was determined by following the changes in the ratio of [^3^H]/[^14^C] expressed as percentage of that of the control untreated with HU mock-silenced cells.

### DNA fiber labeling

DNA fiber analyses were performed as described by Schwab and Niedzwiedz (33) with slight modifications. Exponentially growing PC3 cells were incubated with chlorodeoxyuridine (CldU) and iododeoxyuridine (IdU), their timing and order as indicated in the figures. The first label was used at 50 μM and the second at 250 μM, except for the elongation rate experiments in which the first label (CldU) was used at 25 μM. Spreads were prepared from 4000 cells (suspended in PBS at 2 × 10^6^ cells/ml, ratio of labeled:unlabeled cells = 1:5). Cell lysis was carried out in fiber lysis solution (50 mM EDTA and 0.5% SDS in 200 mM Tris-HCl, pH 7.5). DNA fibers were spread by tilting the slides ∼25° until the drop of the fiber solution reached the bottom of the slide and let to dry. Dried slides were either stored at 4°C or processed immediately. Slides were suspended in 2.5 M HCl for 80 min, washed in PBS, and then incubated in blocking buffer (5% bovine serum albumin in PBS) for 40 min. Primary antibodies—mouse anti-BrdU antibody (Becton Dickinson, cat # 347580) to detect IdU and rat anti-BrdU antibody (Abcam cat# Ab6326) to detect CldU—were diluted in blocking buffer and applied overnight. Slides were washed several times in PBS, incubated with secondary antibodies for 60 min and mounted with ProLong Gold anti-fade reagent (Molecular Probes). Images were acquired with Axiovert 200M microscope (Carl Zeiss) equipped with Axiocam MR3 camera (Carl Zeiss). Fiber length measurements were carried out using AxioVision software (Carl Zeiss).

### Immunofluorescence

For immunofluorescence cells were grown on coverslips, washed in PBS, fixed with 3% formaldehyde in PBS for 5 min at room temperature, permeabilized with 0.5% Triton X-100 in PBS for 5 min, washed again with PBS and blocked in 3% bovine serum albumin in PBS for 1 h. Staining for Rad51 was done using either mouse anti-Rad51 Ab (Ab213, Abcam) diluted 1:100 or rabbit anti-Rad51 Ab (H-92, SantaCruz Biotechnology) using the same dilution overnight at 4°C. To stain for γ-H2AX a rabbit primary antibody (Ab11174, Abcam) diluted 1:100 was used. To stain cells for RPA, coverslips were washed with cytoskeletal (CSK) buffer (10 mM Pipes KOH, pH 6.8; 100 mM NaCl; 300 mM sucrose; 3 mM MgCl_2_; 1 mM EGTA; 0.5% Triton X-100) for 5 min on ice, fixed as above and stained with anti-RPA primary antibody (Ab2175, Abcam) diluted 1:100, overnight. To stain for proliferating cell nuclear antigen (PCNA) cells were also pre-extracted with CSK buffer, fixed with methanol at −20° C and stained with mouse anti-PCNA antibody (PC10, SantaCruz Biotechnology).

Slides were then washed 3 × 5 min in PBS and secondary antibodies were applied. Secondary DyLight 488-conjugated anti-mouse IgG (Abcam) and anti-rabbit secondary IgG DyLight 594 were used at 1:200 dilution for 1 h at room temperature and after 3 × 5 min washes with PBS, slides were mounted using ProLong Gold anti-fade reagent (Molecular Probes).

## RESULTS

### Ino80 depletion sensitizes cells to hydroxyurea and is recruited to chromatin in S phase

**Figure 1.**
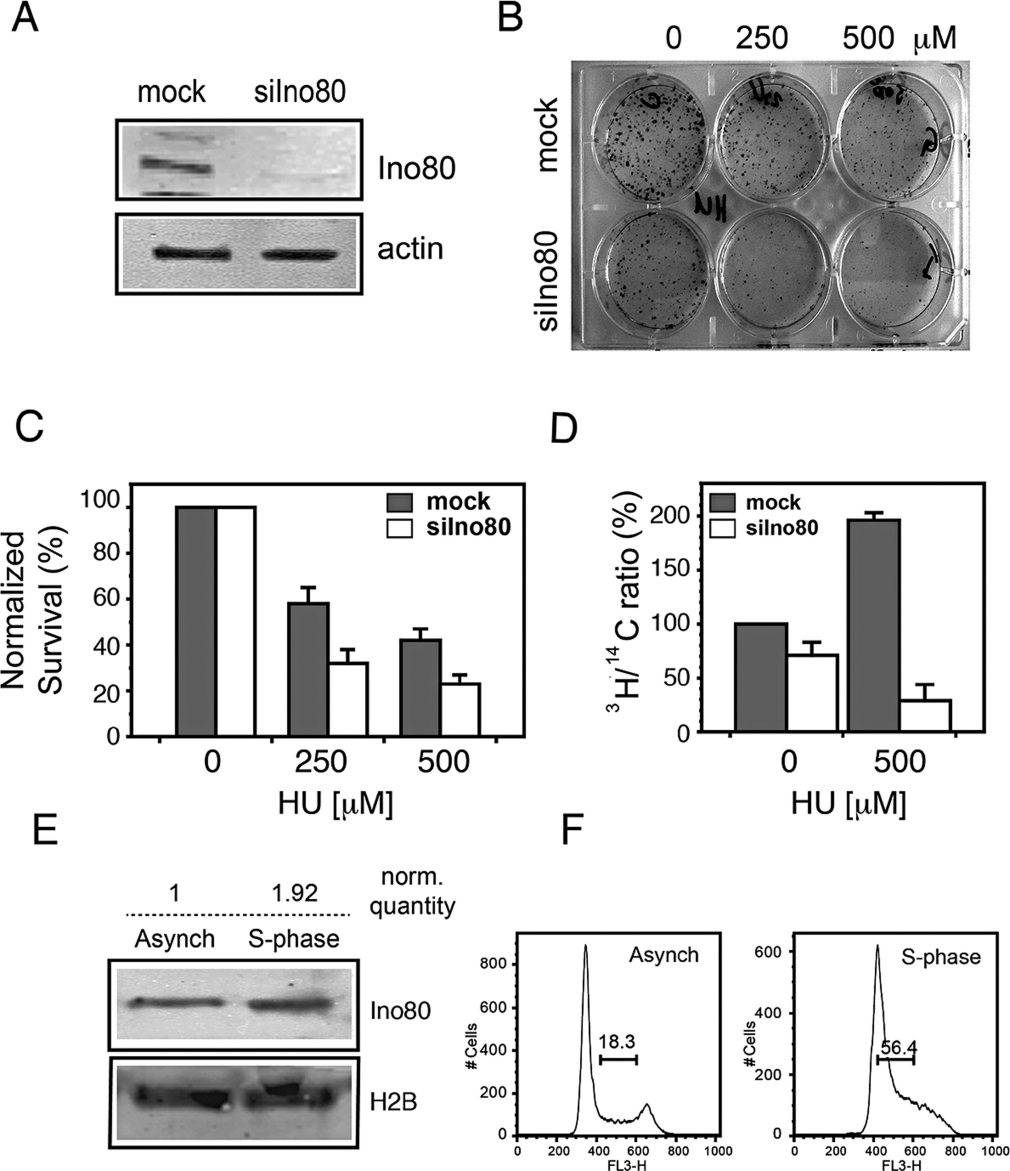
Ino80 sensitizes cells to hydroxyurea. (**A**) Western blot of total extracts from PC3 cells transfected with esiRNAs against GFP (mock) or Ino80 (siIno80) with an antibody against Ino80 3 days after transfection. Actin was used as a loading control. (**B**) Mock and Ino80-silenced cells were seeded at 500 cells/cm^2^, treated with the indicated concentrations of HU for 48 h and left in HU-free medium for 10 days to form colonies. The colonies were then fixed and stained with Gentian violet. (**C**) Clonogenic survival of Ino80-deficient cells after replication stress. Assay was done in triplicate as in (B). Untreated controls are taken as 100%. Data are from three independent experiments, error bars indicate s.d.m. (**D**) Rate of replication in Ino80-silenced cells. Control (mock) and Ino80-depleted cells (siIno80) were labeled with 0.025 μCi/ml ^14^C-dT overnight to uniformly label genomic DNA and were then treated with HU for 6 h or left untreated. After treatment cells were pulse labeled with 1 μCi/ml ^3^H-dT for 30 min. The rate of DNA synthesis was determined by following the changes in the ratio of [^3^H]/[^14^C] expressed as percentage of that of the control untreated with HU mock-silenced cells. (**E**) PC3 cells were collected 12 h after thymidine-nocodazole synchronization and subjected to biochemical fractionation. Equal amounts of the chromatin-bound fraction were analyzed by western blot with an antibody against Ino80. Histone H2B was used as a loading control. Numbers above the lanes indicate the relative quantity of Ino80 after normalization to the loading control. (**F**) Cell cycle profiles of the asynchronous and synchronized cells.

Ino80 is the catalytic subunit of the INO80 chromatin remodeler and also serves as the scaffold of the complex (9). To study the role of Ino80 in replication, we employed RNA interference by endonuclease-prepared small interfering RNA (esiRNA). Because of the complex mixture of many different siRNAs, all targeting the same mRNA, esiRNAs cause effective knockdown of protein expression, while their off-target effects are diluted out (28,30,34). Western blot analysis indicated that 3 days after transfection of human PC3 cells with esiRNA, the Ino80 protein level was greatly reduced in knocked-down compared to control cells (Figure 1A). HU is a potent ribonucleotide reductase inhibitor, which depletes deoxyribonucleotide pools and rapidly and effectively stalls replication forks (35). To assess the sensitivity of Ino80-deficient cells to HU, we used the clonogenic survival assay. Cells were seeded and either left untreated as control or treated with HU for 48 h, washed free of HU and grown for 10 days to form colonies (Figure 1B). The results showed that the knockdown of Ino80 alone diminished viability to about 65% of that of the control (Figure 1B). Replication inhibition by HU reduced colony formation in both mock and Ino80-silenced cells but normalized to the untreated controls, cells with knocked-down expression of Ino80 formed about 40% less colonies (Figure 1C). This indicated that Ino80-depleted cells were more sensitive than the mock-silenced ones and suggested that Ino80 is involved in replication and in replication stress resistance. Therefore, we next assessed the effect of Ino80 knockdown on the rate of DNA synthesis by measuring the incorporation of radiolabeled nucleoside precursor. Under normal conditions, the rate of replication in Ino80-silenced cells was slightly reduced compared to the control (Figure 1D). After release following 6 h in HU, mock-silenced cells displayed a 2-fold increase of incorporation due to partial synchronization in S phase in the presence of the inhibitor. However, in Ino80-deficient cells the rate of DNA replication was more than 5-fold lower (Figure 1D). This decrease strongly suggested that Ino80-deficient cells were defective in recovery from replication stress.

A prerequisite for direct involvement of Ino80 in replication would be the association of this protein with replication forks. To examine whether Ino80 associated with chromatin during S phase, we analyzed the accumulation of the protein in the chromatin-bound fraction using biochemical fractionation. This procedure has been shown to correctly reflect the dynamics of chromatin binding of proteins involved in HR repair (36) and replication (32). Western blot analysis using antibodies against Ino80 revealed a 2-fold increase of the chromatin-bound protein in S-phase cells (Figure 1E) and this increase correlated well with the enrichment of S-phase cells due to synchronization (Figure 1F). These data, together with previous findings (22) suggest that the INO80 chromatin remodeler has a direct function on chromatin during replication.

### Ino80 deficiency leads to impaired replication elongation and increased origin usage

**Figure 2.**
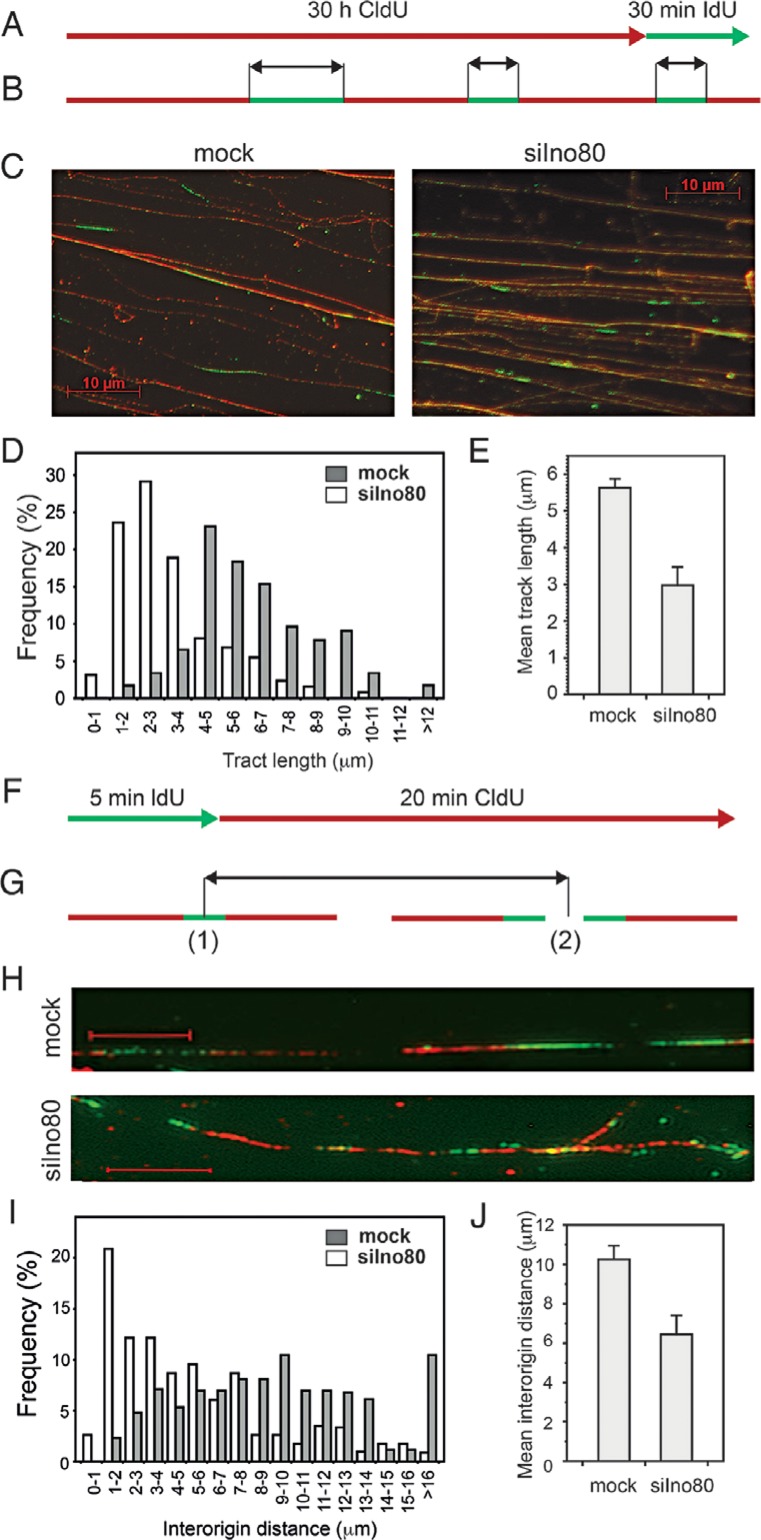
Effect of Ino80 on replication elongation and inter-origin distance. (**A**) PC3 cells were transfected with esiRNAs against GFP (mock) or Ino80 (siIno80). Two days later, mock and Ino80-silenced cells were labeled with CldU for 30 h (>1 cell cycle) to uniformly label genomic DNA (visualized in red) and then pulse labeled with IdU (visualized in green) for 30 min to measure elongation rates. (**B**) A scheme indicating the green tracts, from the 30 min IdU pulse that were measured to estimate elongation rates. (**C**) Representative images of DNA fibers from mock and Ino80-silenced cells. (**D**) Distribution of tract lengths in mock and Ino80-silenced cells following a 30 min IdU pulse. (**E**) Mean tract length in mock and Ino80-silenced cells. Data are from three independent experiments, error bars show s.d.m. At least 150 fibers were measured in each experiment. (**F**) To measure distance between adjacent origins, cells were labeled for 5 min with IdU, and then with CldU for 20 min. (**G**) A scheme of the inter-origin distance measurements: the distance between a newly initiated origin (G.1.) and an origin that has already fired before the first label (G.2.) is shown. Distances between two newly initiated origins (G.1.) or two already initiated ones (G.2.) were also measured. (**H**) Representative images of DNA fibers from mock and Ino80-silenced cells. Scale bar is 5 μm. (**I**) Distribution of inter-origin distances in mock and Ino80-silenced cells. (**J**) Mean inter-origin distance in Ino80-deficient and control cells measured in three independent experiments. Error bars show s.d.m.

To investigate the role of Ino80 at the level of individual replication forks, we measured the rate of elongation and the inter-origin distance in mock and Ino80-silenced cells by fiber labeling. To visualize the DNA fibers, cells were labeled with 25 μM CldU for 30 h. The rate of replication of individual forks was assessed by a 30 min pulse with 250 μM IdU (Figure 2A). This labeling scheme ensured that DNA fiber fragmentation would not complicate the estimation of elongation rates from the green IdU tract measurements (Figure 2B). Immunofluorescent staining of spread fibers (Figure 2C) resulted in green signals from the newly replicated DNA (IdU label) being visible against the background of bulk labeled genomic DNA stained in red (CldU label). Tract length analysis indicated that the elongation rate in Ino80-deficient cells was reduced, compared to the control as the distribution of tract lengths peaked at lower values (Figure 2D). The mean length of the green signal was reduced (Figure 2E) from 5.7 μm in control to 2.9 μm in Ino80-silenced cells, which considering an approximate extension ratio of 2.6 μm/kb (33,37,38) corresponded to replication rates of 0.49 and 0.25 kb/min, respectively. The greater reduction of elongation rates of individual forks, compared to that of bulk replication rate (Figure 1D), suggested that Ino80-deficient cells should fire more origins to replicate their DNA. To assess the effect of Ino80 on replication initiation, we measured the inter-origin distance in mock and Ino80-silenced cells. To this end, cells were labeled with IdU for 5 min, followed by a 20 min pulse with CldU, to establish directionality of replication tracts (Figure 2F). Examination of the distances between origins on individual fibers (Figure 2G and H) indicated that the mean inter-origin distance was reduced in Ino80-deficient cells (Figure 2I and J). Thus, in control mock-silenced cells the mean inter-origin distance was 10.19 μm, while in Ino80-deficient cells it diminished to 6.45 μm. Previous studies have shown that under conditions of replication stress initiation is not inhibited (39) in the already active replication factories (40). The increased replication origin usage in Ino80-deficient cells is consistent with the presence of replication stress in these cells. Taken together, these results indicate that Ino80 is needed for efficient replication elongation, but is dispensable for initiation of new origins under normal conditions.

### Effect of Ino80 on cell cycle distribution and checkpoint signaling

**Figure 3.**
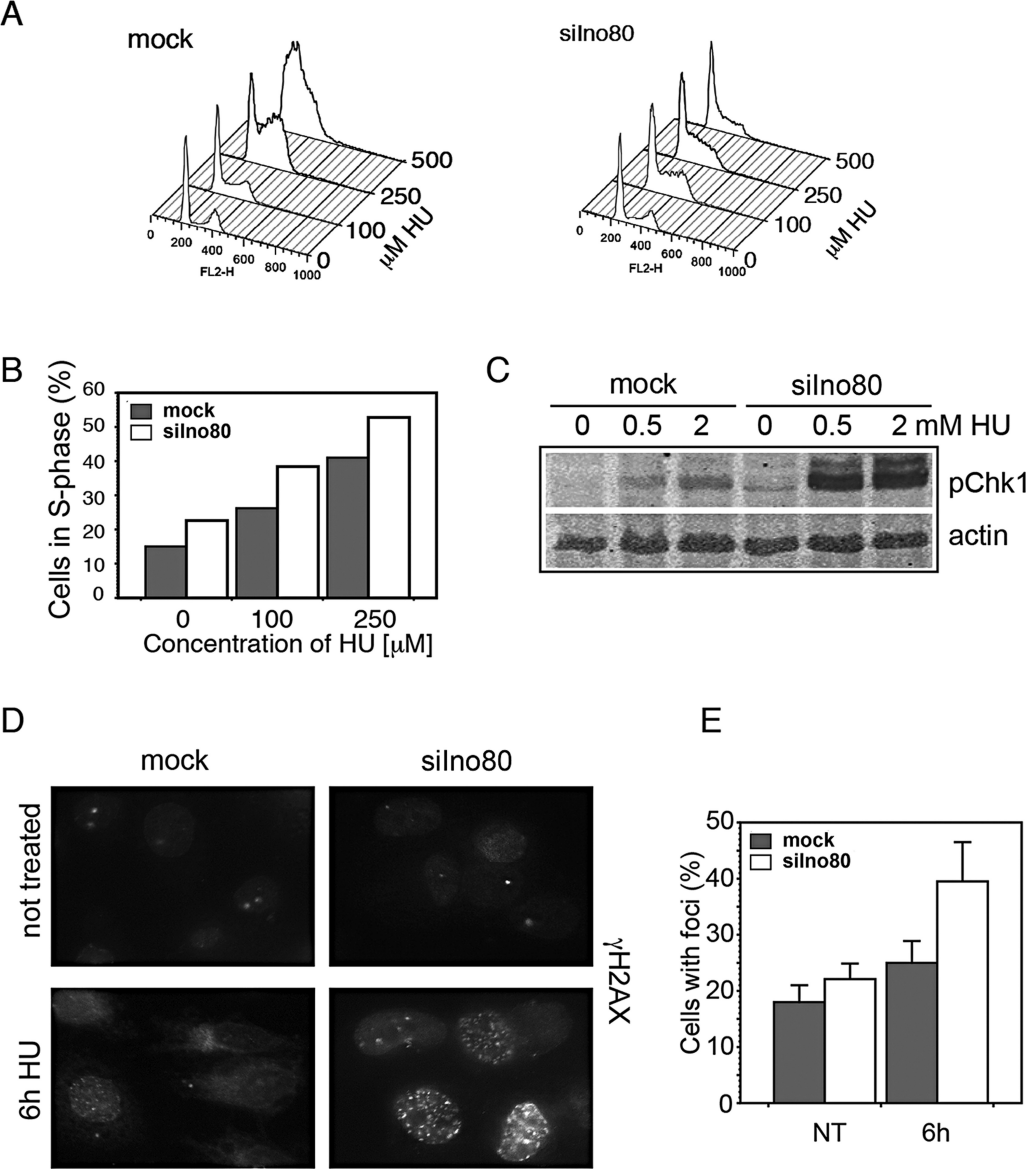
Effect of Ino80 depletion on the cell cycle distribution and checkpoint signaling. (**A**) PC3 cells were transfected with esiRNAs against GFP (mock) or Ino80 (siIno80). Three days later, mock and Ino80-silenced cells were treated with the indicated concentrations of HU for 48 h, fixed, stained with propidium iodide and analyzed by flow cytometry. (**B**) Percentage of S-phase cells in the mock and Ino80-silenced populations at different concentrations of HU as determined in the samples in (A). (**C**) Mock and Ino80-silenced cells were treated with the indicated concentrations of HU for 6 h and phosphorylation of Chk1 on S345 was assessed by western blotting. Actin was used as loading control. (**D**) Mock and Ino80-silenced cells were treated with HU for 6 h, fixed and stained with an antibody against γ-H2AX. (**E**) Percentage of cells with more than 5 γ-H2AX foci.

Since Ino80-deficient cells had lower replication rate and were more sensitive to HU, we analyzed the cell cycle effects of Ino80 depletion. Fluorescence-activated cell sorting (FACS) analysis (Figure 3A) showed that there were 30% more cells in S phase when Ino80 was depleted (Figure 3B). Treatment with increasing concentrations of HU for 48 h resulted in a dose-dependent increase of the S-phase cells but the percentage of S-phase cells remained higher in Ino80-depleted ones than in the mock-silenced controls. At 500 μM HU Ino80-deficient cells failed to progress through the cell cycle (Figure 3A). This accumulation indicated delayed S-phase progression and suggested activation of the S-phase checkpoint. To assess ATR-Chk1 signaling, we followed the level of phopsho-Ser345 Chk1 in mock and Ino80-silenced cells. After 6 h in HU Ino80-deficient cells displayed dramatically higher levels of Chk1 compared with the controls (Figure 3C). The results suggested that in the presence of a replication inhibitor Ino80 depletion caused replication troubles and led to hyperactive S-phase checkpoint.

Histone H2AX is a major substrate of checkpoint kinases - ATR in signaling of replication stress (41) and ATM (42) and DNA-PK (43) in response to DNA double strand breaks. Phosphorylated H2AX (γ-H2AX) in mammalian cells encompasses megabase-sized regions in mammalian cells (44) that could be immunofluorescently visualized as nuclear foci. Immunofluorescence analysis showed that after 6 h in HU, the portion of γ-H2AX positive cells in mock-silenced population was 25%, but increased to 40% in the Ino80-depleted one (Figure 3D and E). The increase of the γ-H2AX positive population, is in line with the hyperphosphorylated status of Chk1. In addition, since γ-H2AX is a major marker of DSBs (Double Strand Breaks) its increase suggests accumulation of inactivated replication forks in Ino80-deficient cells and formation of DSBs.

### Ino80 is needed for the forks to continue after replication inhibition

**Figure 4.**
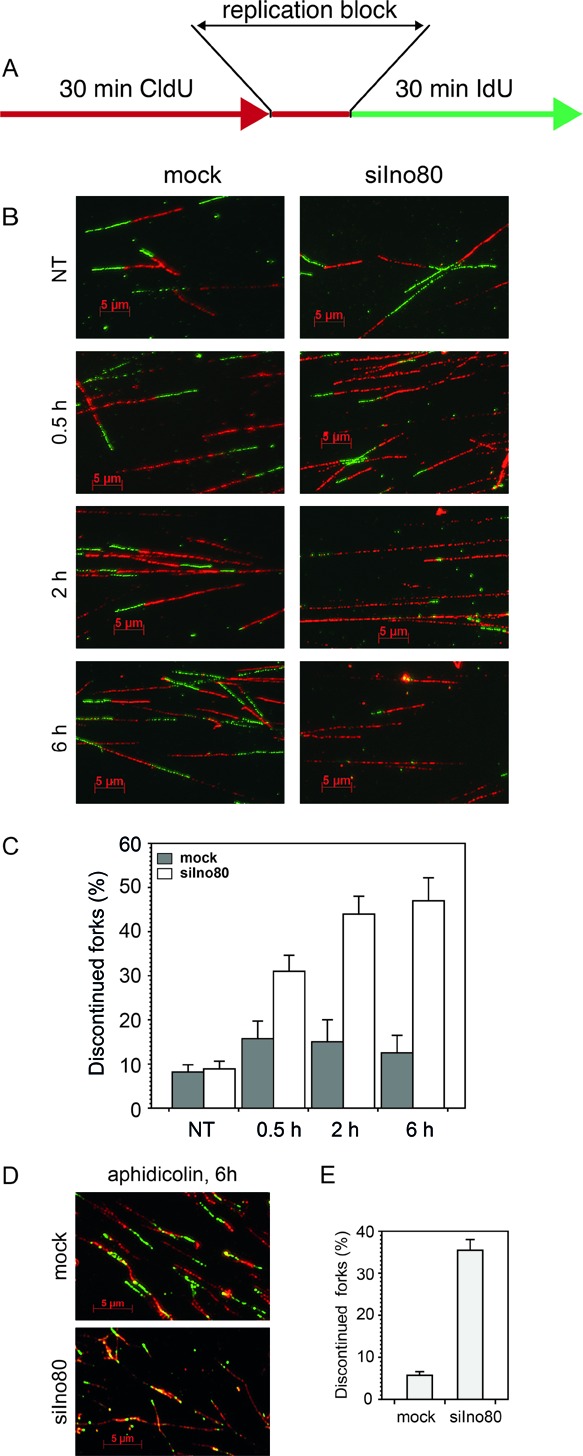
Recovery of stalled replication forks in Ino80-deficient cells. (**A**) To assess fork recovery, mock and Ino80-silenced cells (72 h after transfection) were pulse labeled with CldU for 30 min (red), treated with HU for the indicated times (in the presence of CldU), washed twice with fresh medium and labeled for 30 min with IdU (green). (**B**) Representative images of fibers from cells treated as in (A), times indicate the duration of HU treatment. (**C**) Quantification of discontinued tracts in Ino80-deficient cells treated with HU. The means of three independent experiments are shown, error bars represent s.d.m. At least 150 fibers were measured in each experiment. (**D**) Representative images of fibers after treatment with aphidicolin. (**E**) Quantification of discontinued forks in Ino80-deficient cells treated with aphidicolin for 6 h.

To understand the fate of stalled forks following replication block, we employed a labeling scheme in which after the first label, replication was stalled by HU or aphidicolin, and the second label was added after the release from the replication block (Figure 4A). PC3 cells were labeled with CldU for 30 min and blocked in HU for 0.5, 2 and 6 h (in the presence of CldU). Following treatment, cells were washed free of the replication inhibitor and the CldU and labeled for 30 min with IdU. DNA spreads were prepared (Figure 4B) and the percentage of tracts that contained only the first label (CldU, visualized in red) relative to all replication tracts was determined. While in mock-silenced cells only a small fraction (about 10%) failed to restart after release from HU, in Ino80-silenced cells the percentage of replication tracts that could not be continued increased with the time of HU urea treatment to reach 50% at 6 h in HU (Figure 4B and C). In addition, in Ino80-deficient cells we observed a significant number (that varied between 15 and 20% at different time points) of red tracts terminated with a green dot, suggesting a limited (<0.3 μM, corresponding to 0.8 kb) synthesis after release from the replication block. These structures might have resulted from a less processive incorporation due to various processes (45–47) following fork collapse. Treatment with 2 μM aphidicolin for 6 h produced a similar reduction of the forks that could be restarted (Figure 4D and E). Inability of forks in Ino80-deficient cells to continue replicating after release from HU or aphidicolin indicates that Ino80 is required to maintain stalled forks in a state that permits their restart.

### Consequences of replication stress in Ino80-deficient cells

**Figure 5.**
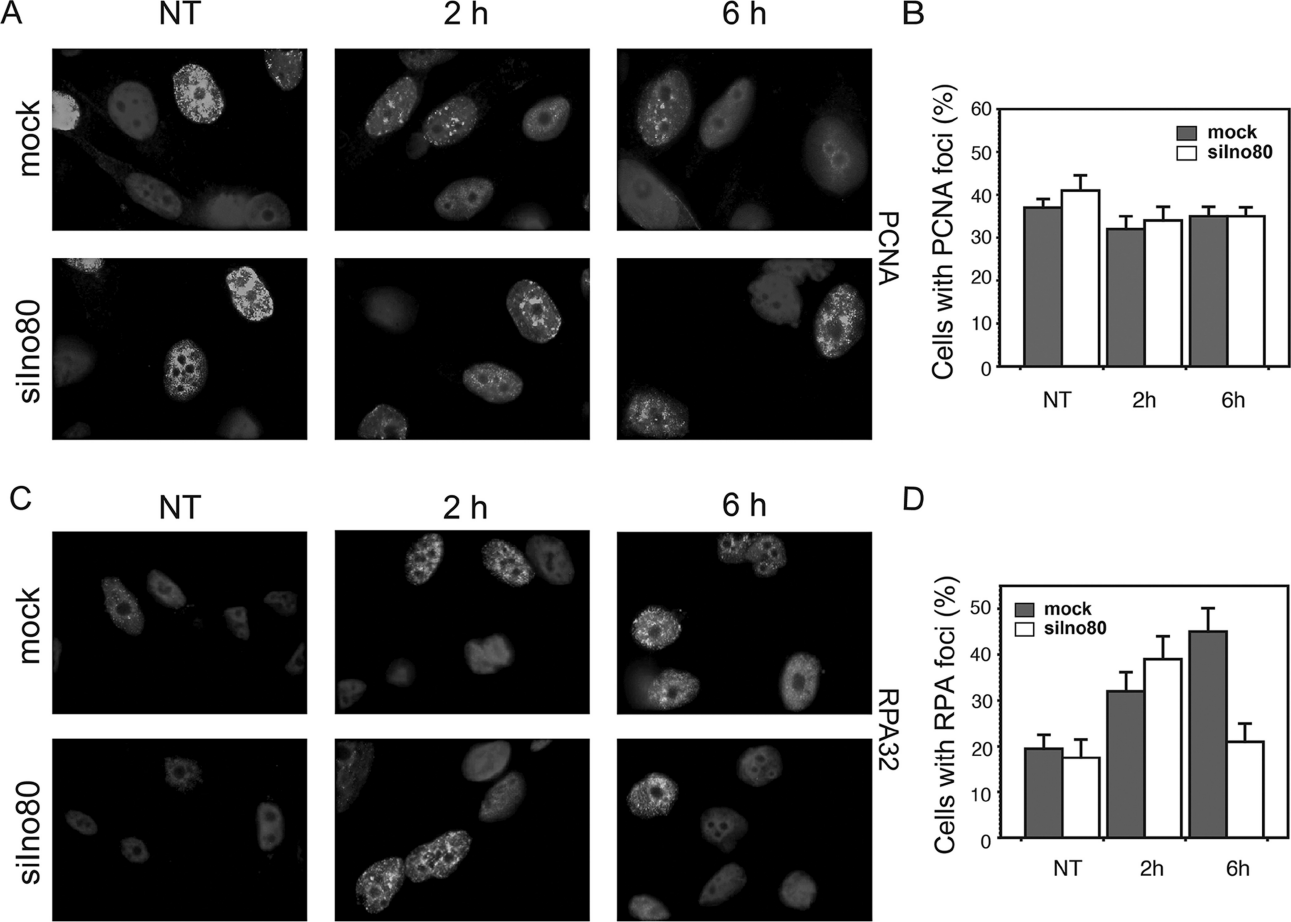
PCNA and RPA foci in Ino80-depleted cells treated with HU. (**A**) PC3 cells were transfected with siRNA against Ino80 or mock-silenced. Seventy two hours later, cells were treated with 0.5 mM HU for the indicated times, pre-extracted with CSK buffer, fixed and stained with an antibody against PCNA. (**B**) The number of PCNA-positive cells was determined in mock- and Ino80-deficient cells treated with HU for 2 and 6 h or left untreated as control (NT). (**C**) PC3 cells were silenced for Ino80 or mock-silenced. Seventy-two hours after transfection, cells were treated as in (A), pre-extracted with CSK buffer, fixed and stained with an antibody against RPA32. (**D**) Quantification of cells containing RPA foci in mock-silenced and Ino80-deficient cells, treated with HU for the indicated times. Data are from three independent experiments, error bars show s.d.m.

A variety of studies have found that if stalled replication forks were not restarted they collapse by losing replisome components and are converted into DSBs (48–50). The increased number of discontinued forks and the increased formation of γ-H2AX foci when Ino80 was depleted suggest that this process is accelerated in deficient cells. To assess the extent to which replisome components are retained on chromatin in Ino80-deficient cells, we followed the nuclear foci formation by PCNA and RPA under conditions of replication stress. Seventy two hours after transfection with esiRNAs, PC3 cells were treated with 0.5 mM HU for 2 and 6 h or left untreated as control. The cells were then pre-extracted, fixed and immunofluorescently stained with an antibody against PCNA. The results indicated that both the size of the PCNA-positive population and the pattern of PCNA foci remained the same in mock and Ino80-silenced cells that were untreated. Treatment with HU for up to 6 h also did not result in significant differences between Ino80-deficient and control cells, though PCNA foci in both were fainter compared with the untreated ones (Figure 5A and B). When stained for RPA, both mock and Ino80-deficient samples contained about 20% of RPA positive cells under normal conditions. Following 2 h in HU, the percentage of RPA positive cells in mock and Ino80-silenced populations increased to about 30–40%, respectively. After 6 h in HU however, the percentage of RPA positive cells in the Ino80-depleted population were reduced to less than half of those in the mock-silenced ones (Figure 5C and D). The decrease of RPA-positive cells indicates that RPA is dissociated from stalled forks when Ino80 is depleted and the cells are under prolonged replication stress.

**Figure 6.**
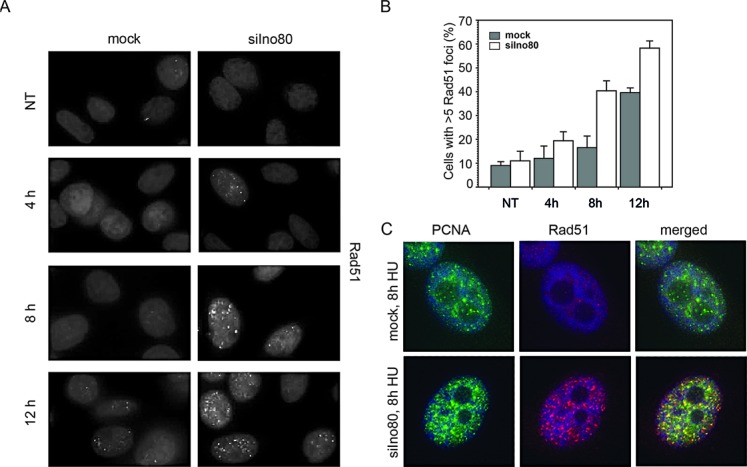
Activation of homologous recombination at replication forks in Ino80-deficient cells treated with HU. (**A**) Control and Ino80-silenced cells (72 h after transfection) were treated with HU for the indicated times, fixed and stained with a Rad51 antibody. (**B**) Percentage of cells with Rad51 foci. (**C**) Colocalization of Rad51 and PCNA foci in cells treated with HU. Mock and Ino80-silenced cells were treated with HU for 8 h, fixed and stained with antibodies against Rad51 (red) and PCNA (green). DNA was counterstained with DAPI (blue).

Rad51 participates in two different pathways of fork restart and repair: stalled forks are restarted in a HR independent manner, while collapsed forks trigger repair by HR. In the recovery from replication stress, Rad51 foci are formed only during HR repair of the collapsed forks (50). Mock and Ino80-silenced cells were treated with 0.5 mM HU for 4, 8 and 12 h and were then stained with a Rad51 antibody (Figure 6A). The obtained results indicated a time-dependent increase of cells with Rad51 foci that was much faster when Ino80 was knocked-down and after 8 h in HU, there was a more than 2-fold increase of the cells containing Rad51 foci in the Ino80-deficient population relative to the control one (Figure 6A and B). It should be noted that the significant increase of Rad51 foci formation occurred after the decrease of the RPA signal. To confirm that Rad51 foci were formed at replication sites, we performed co-staining of Rad51 with PCNA. The results showed that the Rad51 signal co-localized with PCNA foci (Figure 6C). Altogether these results indicate that in the absence of Ino80, HR repair is triggered as replication forks get inactivated and damaged.

### Effect of Arp8 depletion on recovery after replication stress

To understand whether the participation of Ino80 in recovery after replication stress was specific to the protein or a characteristic function of the whole INO80 complex we knocked-down expression of Arp8 (Supplementary Figure S1A) and evaluated the effect of its depletion on the recovery of stalled forks and on γ-H2AX foci formation. To determine the recovery of stalled replication forks, mock and Arp8-silenced cells were labeled with halogenated nucleosides as outlined in Figure 4A. Fiber labeling analysis of replication tracts that continue after 6 h in HU indicated a 4-fold increase of discontinued forks (Supplementary Figure S1B and C). As expected, immunofluorescent staining of Arp8-depleted cells after 6 h in HU showed a nearly 2-fold increase of γ-H2AX positive cells (Supplementary Figure S1D and E). Thus, depletion of the Arp8 subunit had the same consequences as Ino80 deficiency. The fact that depletion of two unique subunits of INO80 exhibited similar outcomes suggests that the remodeler as a complex is required for stabilization of stalled replication forks.

## DISCUSSION

This work aimed to examine the requirements for the human INO80 chromatin remodeler in both unperturbed replication and in recovery from replication stress, motivated by the limited data of its participation in DNA replication in mammalian cells and the disparate conclusions reached by yeast studies. To elucidate the mechanism of action of INO80 in DNA replication, we systematically investigated the involvement of the complex during unchallenged replication and under conditions of replication stress by following the effects of its depletion on cell survival, the fate of individual replication forks, S-phase checkpoint activation and the consequences of prolonged fork stalling. Thus by using the fiber labeling technique for the first time we showed that the mammalian remodeler was specifically needed for replication elongation but was dispensable for initiation of replication during unperturbed S phase. Fiber labeling experiments in Ino80-deficient cells demonstrated shorter replication tracts (Figure 2 C, D and E) but more closely spaced origins of replication (Figure 2 H, I and J), indicating the need for initiation of extra origins to complete S phase. This supports the results in yeast that although Ino80 was recruited to a significant portion of the yeast autonomous replication sequences, INO80 was dispensable for initiation of replication (23,25). Further, for the first time in mammalian cells, we addressed the role of the INO80 complex in replication stress. Fiber labeling experiments performed with two inhibitors of DNA synthesis (HU and aphidicolin) provided evidence indicating that mammalian INO80 stabilized stalled replication forks and permitted their restart following release from replication arrest. While in mock-silenced cells less than 10% of replication forks failed to restart after release from the replication block, in Ino80-silenced cells about half of the forks could not be continued (Figure 4). These results are in line with the data obtained from yeast experiments that the INO80 chromatin remodeler stabilizes stalled replication forks and ensures fork resumption after release from replication arrest (23,25) and do not support the data showing that INO80 is not required to stabilize and restart stalled replication forks (24).

Important elements of our systematic study on the role of INO80 in DNA replication in mammalian cells was the examination of γ-H2AX, Rad51, RPA and PCNA foci formation. The results showed that after exposure to HU, cells knocked-down for Ino80 accumulated γ-H2AX and Rad51 foci (Figure 3D, E and Figure 6) These observations are in line with the data that yeast Ino80 mutants suffered significantly impaired fork resumption and accumulated the HR repair protein Rad52 after HU treatment (23). We also observed a reduction of RPA positive cells after prolonged treatment with HU in Ino80-deficient cells. While control and Ino80-deficent cells displayed similar percentage of RPA positive cells after 2 h in HU, the percentage of Ino80-depleted cells with RPA foci declined twice relative to the control after 6 h under replication stress. At the same time, PCNA foci did not follow such dynamics, as these were comparable in control and Ino80-deficient cells (Figure 5). The RPA foci reduction in Ino80-deficient cells most likely was a consequence of the dissociation of replication fork components following replication fork collapse. This is a similar outcome to what has been reported in an yeast Ino80 deletion strain, in which replication inhibition resulted in dissociation of DNA pol α and RPA, but not PCNA (25). As time course experiments showed that the increase of Rad51 foci formation occurred after the decrease of the number of RPA foci, the reduction of the RPA signal might also reflect active displacement of RPA by Rad51 as HR repair of collapsed forks proceeds. Such a phenomenon has been reported in yeast checkpoint mutant cells (and thus unable to stabilize stalled forks), which after release in S-phase in the presence of HU, failed to accumulate RPA at the earliest origins of replication (51), but these sequences were bound by Rad52 (52).

The next observation in this study that should be discussed is the activation of the S-phase checkpoint in Ino80-depleted cells. Incubation of the ino80-depleted cells with HU led to robust activation the checkpoint kinase (Figure 3C). Data in yeast have shown that following treatment with HU, ino80Δ cells activated the replication checkpoint and permanently arrested with high Rad53 kinase activity as they were unable to complete DNA synthesis (25). Another study have reached to a different conclusion—that yeast INO80 and ISW2 remodelers control the deactivation of the S-phase checkpoint, but do not participate in replication fork protection (26). However, our data (presented on Figures 4, 5 and 6) support the view that in mammalian cells the hyperphosphorylation of Chk1 in Ino80-deficient cells is not the primary effect of Ino80 depletion, but rather a consequence of decreased fork stability, fork collapse and formation of double-strand breaks. This was further supported by the observation that simultaneous knockdown of Ino80 and Chk1 produced even stronger replication defects, suggesting that the two proteins act by different mechanisms (not shown). Thus, our data outline a mechanism of action of INO80 in mammalian DNA replication, in which the remodeler stabilizes stalled replication forks, permits their restart following release from replication arrest, attenuates S-phase checkpoint activation and prevents double-strand breaks formation and subsequent HR. Since the INO80 complex is a transcription factor, one possibility would be that the reduction of fork stability could be due to transcriptional deregulation of replisome components. However, gene expression profiling in yeast has provided strong evidence that the DNA repair and replication functions of yeast Ino80 were not due to transcriptional defects (13,53). Additionally, yeast Ino80 is recruited to replication forks in yeast under normal conditions and when stalled (23–25), which argues for a direct role of the complex there. In mammalian cells, our data and that of others indicate its recruitment to chromatin in S phase (Figure 1E) (22), suggesting direct involvement of the complex. Given that deficient cells are simultaneously defective in replication elongation and fork restart, it could be that the complex directly regulates the balance of replication fork components involved in either DNA replication or histone turnover and thus facilitates fork stability. It is also possible that INO80 remodeler may influence chromatin environment in a way that assists other DNA-templated processes. Thus, it has been proposed that mislocalization of histone H2AZ has an inhibitory effect on genome integrity (54), probably due to the lower turnover of H2AZ-containing nucleosomes (55,56). An important challenge for the future would be to find out the role of mammalian INO80 in chromatin replication and the major reorganization of nucleosomes during this process.

## SUPPLEMENTARY DATA

Supplementary Data are available at NAR Online.

SUPPLEMENTARY DATA
