# Supplementary material for: The mammalian INO80 chromatin remodeling complex is required for replication stress recovery
Source: Nucleic Acids Res. 2014 Jul 12;42(14):9074–86. doi: 10.1093/nar/gku605 (PMC4132725; doi:10.1093/nar/gku605)
Supplement: SUPPLEMENTARY DATA [file supp_42_14_9074__index.html]

The mammalian INO80 chromatin remodeling complex is required for replication stress recovery — The mammalian INO80 chromatin remodeling complex is required for replication stress recovery — SUPPLEMENTARY DATA 

# The mammalian INO80 chromatin remodeling complex is required for replication stress recovery

## SUPPLEMENTARY DATA

**Files in this Data Supplement:**

- SUPPLEMENTARY DATA
- SUPPLEMENTARY DATA
