# Supplementary figures and images for: The mammalian INO80 chromatin remodeling complex is required for replication stress recovery
Source: Nucleic Acids Res. 2014 Jul 12;42(14):9074–86. doi: 10.1093/nar/gku605 (PMC4132725; doi:10.1093/nar/gku605)

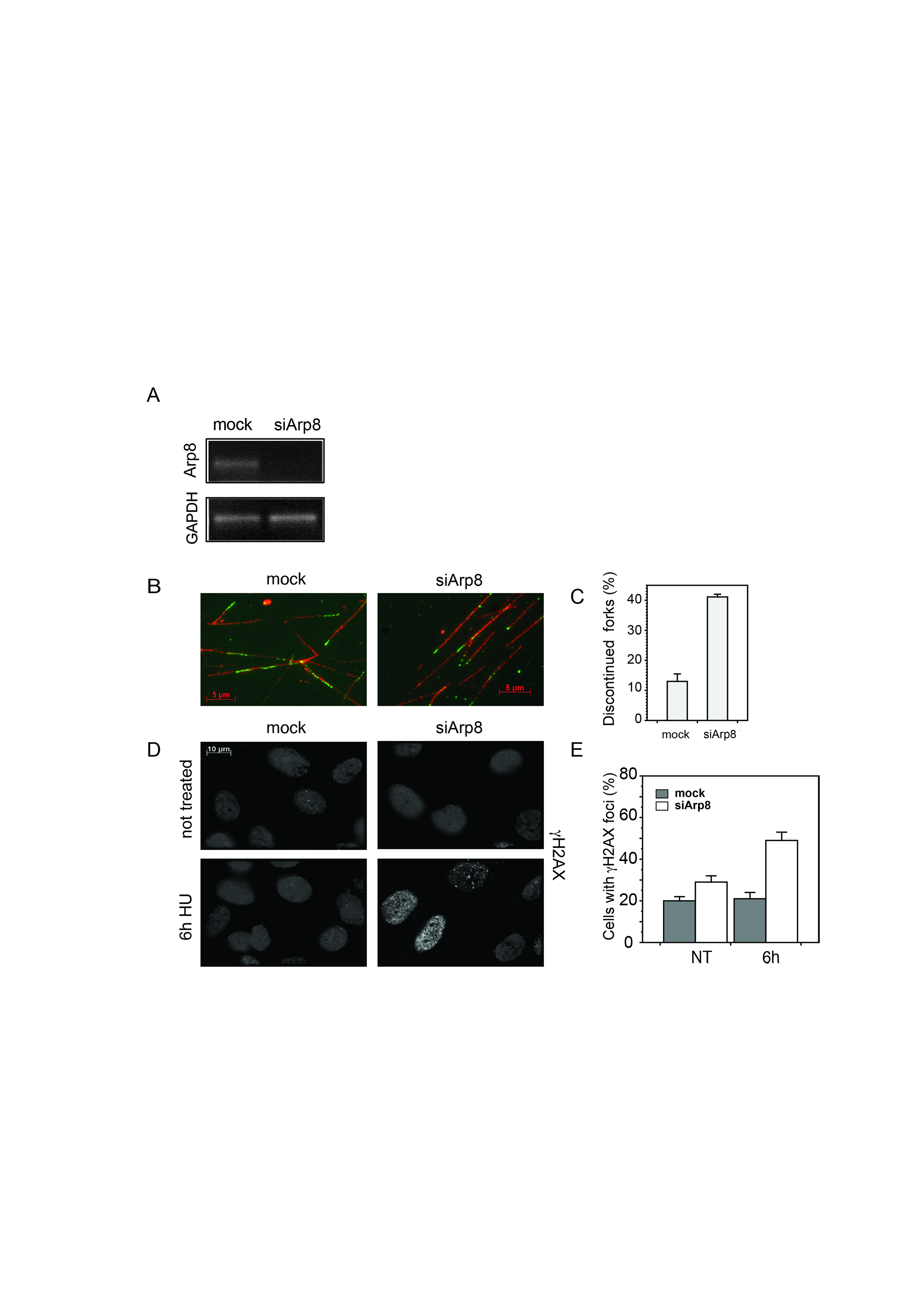

Supplement: SUPPLEMENTARY DATA [file supp_gku605_nar-03386-m-2013-File007.tif]
