# Supplementary material for: The mammalian INO80 chromatin remodeling complex is required for replication stress recovery
Source: Nucleic Acids Res. 2014 Jul 12;42(14):9074–86. doi: 10.1093/nar/gku605 (PMC4132725; doi:10.1093/nar/gku605)
Supplement: SUPPLEMENTARY DATA [file supp_gku605_nar-03386-m-2013-File009.docx]

Supplementary Fig. 1. Arp8-deficient cells are compromised in fork recovery after replication stress. (A) PC3 cells were silenced for 72 hours with esiRNA against Arp8 or GFP as control and the levels of Arp8 mRNA were determined by RT-PCR. (B) Mock and Arp8-silenced cells were pulse labeled with CldU for 30 min (red), treated with 0.5 mM HU for 6h (in the presence of CldU), washed twice with fresh medium and labeled for 30 min with IdU (green). Representative images of the spread fibers from control and Arp8-silenced cells are given. (C) Quantification of discontinued forks in Arp8-deficient and control cells after treatment with HU. The means of 3 independent experiments are shown, error bars represent s.d.m. At least 150 fibers were measured in each experiment. (D) Mock and Arp8-deficient cells were treated with 0.5 mM of HU for 6h, fixed and stained with an antibody against γ-H2AX. (E) Percentage of cells with more than 5 foci.
